# Wide-field fluorescein and indocyanine green angiography findings in the eyes with Vogt-Koyanagi-Harada disease

**DOI:** 10.1186/s12348-017-0134-3

**Published:** 2017-07-11

**Authors:** Ryo Kurobe, Yoshio Hirano, Naomi Niwa, Kazuhiko Sugitani, Tsutomu Yasukawa, Munenori Yoshida, Yuichiro Ogura

**Affiliations:** Department of Ophthalmology & Visual Science, Nagoya City University Graduate School of Medical Sciences, 1-Kawasumi, Mizuho-cho, Mizuho-ku Nagoya, 467-8601 Japan

**Keywords:** Wide-field indocyanine green angiography, Densitometry, Binarization, Vogt-Koyanagi-Harada disease, Peripheral lesion

## Abstract

**Background:**

The purpose of this study is to report wide-field angiography findings before and after steroid therapy in a case with bilateral Vogt-Koyanagi-Harada (VKH) disease.

**Results:**

A 44-year-old woman presented with bilateral blurred vision and metamorphopsia accompanied by symptoms of headache and tinnitus. The baseline best-corrected visual acuity was 20/20 in both eyes. Ophthalmic examination revealed a shallow anterior chamber and panuveitis accompanied by multiple serous retinal detachments in both eyes and ciliochoroidal detachments in the left eye. Wide-field fluorescein angiograms showed hyperfluorescene indicating pooling corresponding to multiple serous retinal detachments in the posterior lesion and vascular leakage in the peripheral retina and choroid, resolved after steroid tapering therapy. Interestingly, wide-field indocyanine angiograms revealed narrowing of choroidal vessels in the acute phase and its normalization with resolution of inflammation after the therapy.

**Conclusions:**

Eyes with Vogt-Koyanagi-Harada disease had peripheral chorioretinal vascular leakage and choroidal vessel narrowing in the acute phase. Wide-field angiography is a useful tool to reveal peripheral chorioretinal findings and assess diameters and density of choroidal vessels.

## Findings

Vogt-Koyanagi-Harada (VKH) disease is defined as a bilateral glanulomatous panuveitis with or without extraocular manifestations such as meningismus, tinnitus, perception deafness, cerebrospinal fluid pleocytosis, alopecia, poliosis, and depigmentation of the skin [1]. In the acute phase, several widespread ocular manifestations including swelling of the optic disc, multiple serous retinal detachments, thickening of the choroid, and ciliochoroidal detachments were observed [1, 2]. Adequate doses of corticosteroids soon after the disease onset often resolve the ocular and systemic manifestations [3]. Several previous reports [2, 4] have shown fluorescein angiography (FA) and indocyanine green angiography (ICGA) findings in the eyes with VKH disease. However, no reports described peripheral findings in the eyes with VKH disease using wide-field FA and ICGA. In the current case report, we evaluated the wide-field FA and ICGA findings before and after steroid therapy in the eyes with VKH disease as well as the clinical observations.

## Case report

**Fig. 1:**
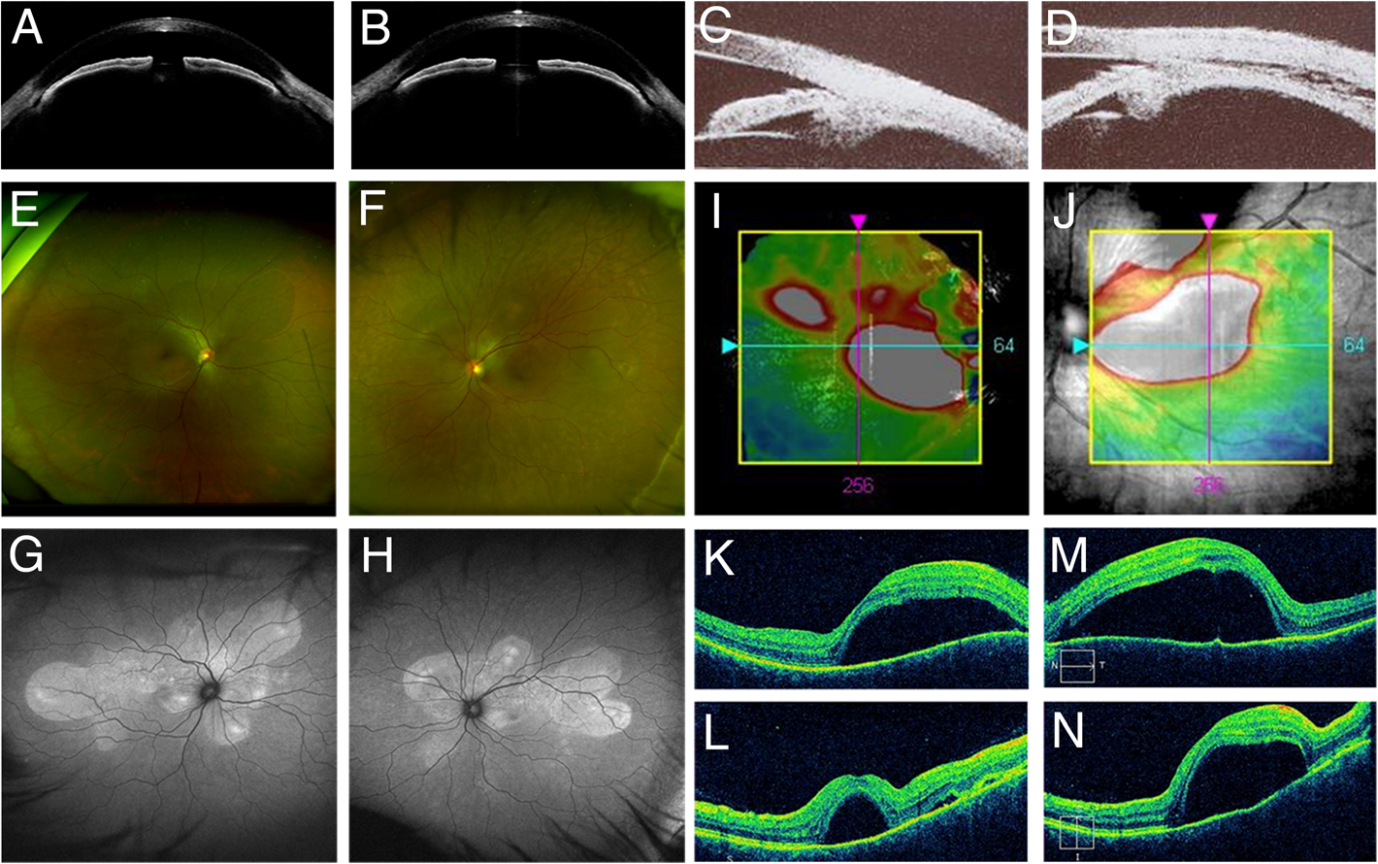
Baseline findings. **a**, **b** Anterior segment optical coherence tomography findings. **a** Right eye. **b** Left eye. The anterior chamber was shallow, and the angle was narrow in both eyes. The depth of the anterior chamber was 1.42 and 1.65 mm in the right and the left eyes, respectively (**a–b**). **c**, **d** Ultrasound biomicroscopy findings. **c** Right eye. **d** Left eye. A ciliochoroidal detachment was observed in the left eye (**d**). **e**, **f** Fundus photography findings. **e** Right eye. **f** Left eye. Multiple serous retinal detachments were observed in both eyes. **g**, **h** Fundus autofluorescence findings. **g** Right eye. **h** Left eye. Hyperautoflurescence consistent with the serous retinal detachments were observed in both eyes. **i**–**n** Optical coherence tomography findings. **i**, **j** Color map. **k**, **m** Horizontal. **l**, **n** Vertical images. **i**, **k**, **l** Right eye. **j**, **m**, **n** Left eye. Multiple serous retinal detachments were observed in both eyes

**Fig. 2:**
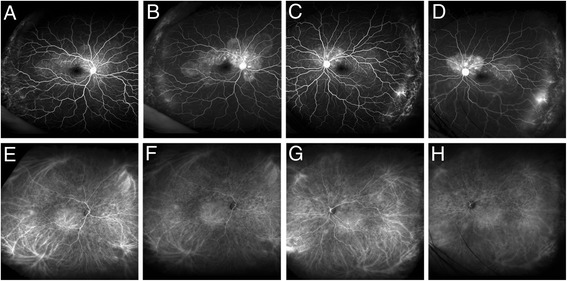
Baseline wide-field angiography findings using Optos California. **a**–**d** Fluorescein angiograms. **a** Early phase image in the right eye. **b** Late phase image in the right eye. **c** Early phase image in the left eye. **d** Late phase image in the left eye. Hyperfluorescence indicating pooling due to multiple serous retinal detachments in the posterior lesions and hyperfluorescence due to leakage in the temporal periphery were observed in both eyes. **e**–**h** Indocyanine green angiograms. **e** Early phase image in the right eye. **f** Late phase image in the right eye. **g** Early phase in the left eye. **h** Late phase image in the left eye. Hyperfluorescence indicating leakage from the choroidal vessels in the peripheral lesions and multiple hypofluorescent dots were observed in both eyes

**Fig. 3:**
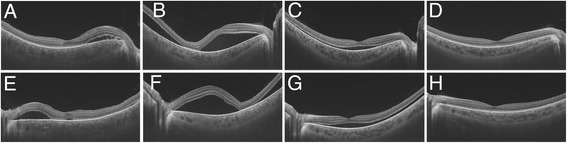
High-penetrating swept-source optical coherence tomography findings during the follow-up. **a**–**d** Right eye. **e**–**h** Left eye. **a**, **e** Baseline. **b**, **f** One week after the therapy. **c**, **g** One month after the therapy. **d**, **h** Three months after the therapy. High-penetrating swept-source optical coherence revealed that serous retinal detachments resolved accompanied with a decrease of the central choroidal thickness in both eyes

**Fig. 4:**
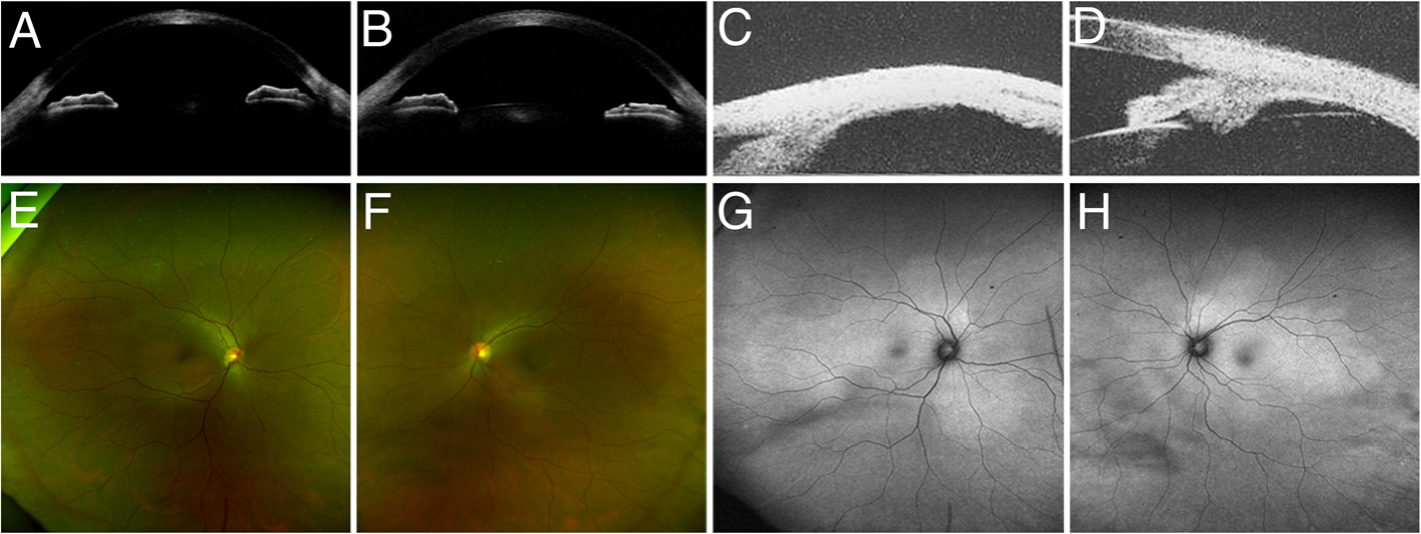
Findings 7 months after the steroid tapering therapy. **a**, **b** Optical coherence tomography findings. **a** Right eye. **b** Left eye. The anterior chamber got deeper, and the angle was open in both eyes. The depth of anterior chamber recovered to 3.00 and 2.93 mm in the right and left eyes, respectively. **c**, **d** Ultrasound biomicroscopy findings. **a** Right eye. **b** Left eye. **d** The ciliochoroidal detachment in the left eye completely resolved. **e**, **f** Fundus photography findings. **e** Right eye. **f** Left eye. The multiple serous retinal detachments disappeared in both eyes. **g**, **h** Fundus autofluorescence findings. **g** Right eye. **h** Left eye. The hyperautofluorescence became faint in both eyes

**Fig. 5:**
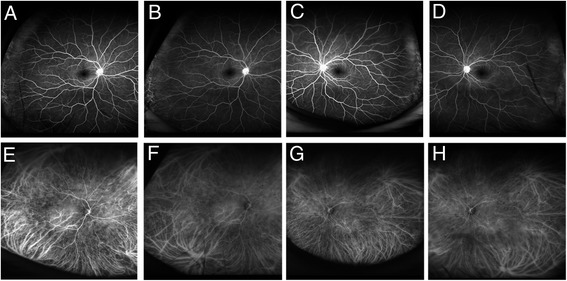
Wide-field angiography findings using Optos California 7 months after the steroid tapering therapy. **a**–**d** Fluorescein angiograms. **a** Early phase image in the right eye. **b** Late phase image in the right eye. **c** Early phase image in the left eye. **d** Late phase image in the left eye. The hyperfluorescence at baseline almost resolved in both eyes. **e**–**h** Indocyanine green angiograms. **e** Early phase image in the right eye. **f** Late phase image in the right eye. **g** Early phase image in the left eye. **h** Late phase image in the left eye. The hyperfluorescence at baseline almost resolved in both eyes, but the hypofluorescent dots still remained in both eyes

A 44-year-old woman presented with bilateral blurred vision and metamorphopsia accompanied by symptoms of headache and tinnitus. The best-corrected visual acuity (BCVA) was 20/20 in both eyes, and the intraocular pressure (IOP) was 20 and 23 mmHg in the right and left eyes, respectively. The spherical equivalent (SE) was −6.25 and −6.0 diopter (D) in the right and left eyes, respectively. Ophthalmic examination revealed conjunctival hyperemia and inflammatory cells in the anterior chamber and vitreous cavity in both eyes. Anterior segment swept-source optical coherence tomography (SSOCT) (SS-1000 CASIA, Tomey, Nagoya, Japan) revealed that the anterior chamber was shallow and the angle was narrow in both eyes (Fig. 1). The axial length measured with IOL master 500 (Carl Zeiss Meditec, Dublin, CA) was 23.53 and 23.40 mm in the right and left eyes, respectively. Ultrasound biomicroscopy (UBM) (UD-1000, Tomey, Nagoya, Japan) revealed a ciliochoroidal detachment in the left eye (Fig. 1). Color fundus photographs and fundus autofluorescence images revealed multiple serous retinal detachments and hyperautofluorescence consistent with the area of serous retinal detachments (Fig. 1). Optical coherence tomography (OCT) (Cirrus HD-OCT, Carl Zeiss Meditec, Dublin, CA) images from both eyes revealed multiple serous retinal detachments (Fig. 1). Using Optos California (Optos plc, Dunfermline, Scotland), wide-field retinal imaging device, FA images showed hyperfluorescence indicating pooling corresponding to multiple serous retinal detachments in the posterior lesion and chorioretinal vascular leakage in the peripheral lesion (Fig. 2). Hyperfluorescence at the optic discs in both eyes also was observed (Fig. 2). Interestingly, Optos California wide-field ICGA images revealed narrowing of vessels in the acute phase as well as multiple hypofluorescent dark dots and hyperfluorescence due to leakage from choroidal vessels (Fig. 2). Spinal fluid examination showed evidence of pleocytosis (polynuclear leukocyte 2, mononuclear leukocyte 39). Also, both human leukocyte antigen DR4 and DQ4 were positive. The patient was diagnosed as a VKH disease and received systemic corticosteroids tapering therapy for more than 6 months and eye drops of betamethasone sodium phosphate. Steroid tapering therapy was performed as follows: 1000 mg of methylprednisolone for 3 days, 40 mg of prednisolone for 2 weeks, 30 mg of prednisolone for 1 month, 25 mg of prednisolone for 1 month, 20 mg of prednisolone for 1 month, 15 mg of prednisolone for 1 month, 10 mg of prednisolone for 1 month, 7.5 mg of prednisolone for 1 month, 5 mg of prednisolone for 2 weeks, and 2.5 mg of prednisolone for 2 weeks. After an administration of systemic steroid therapy, the serous retinal detachments in both eyes gradually resolved accompanied with a decrease of the central choroidal thickness and completely disappeared 3 months after the start of therapy (Fig. 3). Seven months after the start of steroid therapy, the BCVA improved to 20/13 and the IOP returned to normal in both eyes. The SE recovered to −3.75 and −3.25 D in the right and left eyes, respectively. The axial length after the therapy was 23.50 and 23.32 mm in the right and left eyes, respectively, and both of which were unchanged compared to those at baseline. Figure 4 shows the inflammation almost resolved after the therapy. On wide-field FA images, the hyperfluorescence due to multiple serous retinal detachments almost disappeared, but the hyperfluorescence at the optic disc still remained (Fig. 5). On wide-field ICGA images, the hyperfluorescence indicating leakage from choroidal vessels almost disappeared, but the multiple hypofluorescent dark dots still remained (Fig. 5).

## Binarization of wide-field indocyanine green angiography images

**Fig. 6:**
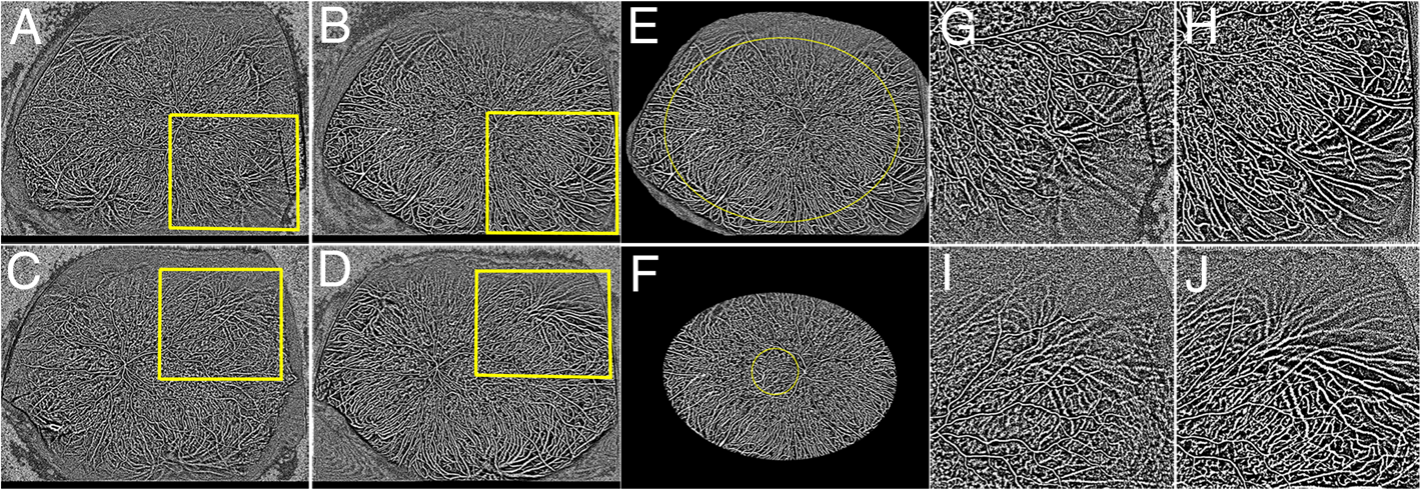
Binarized images of wide-field indocyanine green angiograms before and after treatment. **a** Baseline image in the right eye. **b** Posttreatment image in the right eye. **c** Baseline image in the left eye. **d** Posttreatment image in the left eye. **e**, **f** Densitometry of retinal and choroidal vessels. **e** The same image as **b**. The *yellow oval* is drawn through one-disc diameter posterior to the center of the ampullas of vortex veins. **f** Trimmed image along the *yellow oval* on **e**. The *yellow circle* indicates posterior area within 3 mm from the fovea and the outside of that was considered as the mid-periphery. **g**, **h** Magnified images of the *yellow square* in **a** or **b**.**i**, **j** Magnified images of the *yellow square* in **c** or **d**. After the therapy, the choroidal vessels were clearly visualized and the narrowed vessels in the acute phase resolved in both eyes

We previously reported binarization technique of wide-field ICGA images in the eyes with central serous chorioretinopathy [5], leading to a possibility of densitometry of choroidal vessels in wider area. In this current report, the same technique was applied to evaluate a densitometry of choroidal vessels before and after steroid therapy in the eyes with VKH disease. However, a subtraction of fluorescein signals from ICGA images have failed because of intensive subretinal pooling of fluorescein dye. Therefore, the densities of retinal and choroidal vessels in both posterior and mid-peripheral lesions were compared between baseline and posttreatment (Fig. 6) using the method reported previously [5]. The densities are shown in Table 1. In both the posterior and mid-peripheral lesions, the densities at baseline were lower than after the treatment in both eyes. Figure 6 shows the binarized images of ICGA. After the therapy, choroidal vessels were clearly visualized and the narrowed vessels recovered in the peripheral lesions (Fig. 6).

**Table 1:** Densities of retinal and choroidal vessels

Eye	Time of measurement	Posterior	Mid-peripheral
Right eye	Baseline	36.5%	36.2%
	Posttreatment	38.1%	37.9%
Left eye	Baseline	34.7%	35.6%
	Posttreatment	35.6%	36.0%

## Discussion

In the current case report, we performed wide-field FA and ICGA using Optos California before and after steroid therapy in the eyes with VKH disease, which revealed peripheral chorioretinal lesions and enabled densitometry of retinal and choroidal vessels by binarization of wide-field ICGA images. We found that peripheral findings as well as posterior findings were observed in this patient. Recent reports described that wide-field imaging was useful to identify peripheral lesions in diabetic retinopathy (DR) [6] and age-related macular degeneration [7, 8] and assess progression of diseases such as DR and retinitis pigmentosa [6, 9]. There are no reports describing wide-field angiography findings in the eyes with VKH disease. However, several reports [10–12] described ciliochoroidal detachments using UBM the same as in the current patient, indicating that the peripheral lesions also were involved in the eyes with VKH disease. Therefore, wide-field angiography was useful to evaluate the wider area than conventional FA and ICGA.

We previously reported [5] that binarization of wide-field ICGA images enabled quantification of choroidal vessel density in the eyes with central serous chorioretinopathy. In the current report, the same method was used to evaluate the change before and after the therapy in the eyes with VKH disease. Although a subtraction of FA images from the ICGA images had failed because of intensive hyperfluorescent signals related to subretinal pooling of fluorescein dye, we could recognize the decreased densities using binarization of wide-field ICGA in the acute phase and those recoveries after the therapy in both posterior and peripheral lesions. Kim et al. [13] reported that parafoveal capillary density using OCT angiography images in the eyes with uveitis was significantly lower than in the healthy controls. In addition, Hirooka et al. [14] reported that macular mean blur rate using laser speckle flowgraphy significantly increased after treatment accompanied with a decrease of central choroidal thickness in the eyes with VKH disease. Taken together, in the eyes with VKH disease, the inflammation causes vascular hyperpermeability and diffuse infiltration of lymphocytes and fluids into the choroidal stroma, resulting in a marked choroidal thickening [14]. Then, the choroidal vessels are compressed by the increased hydrostatic pressure in the choroidal stroma and the blood flow might be decreased [14]. In the current case, the densities in both the posterior and peripheral lesions in the acute phase were lower than those after the therapy. Future studies with more samples should be needed to confirm this result and elucidate underlying pathogenesis.

The current study has several limitations. First, this is just a case report. With large samples, a quantification of choroid vessels density by which fluorescein signals were subtracted from the ICGA images should be examined. Second, there were no appropriate controls, which means age-matched controls with neither any inflammation nor retinal or choroidal diseases.

In conclusion, the current case report showed that wide-field angiograms revealed not only that peripheral lesion was involved in the eyes with VKH disease but also that the vessel diameters and the vessel density in the peripheral lesion decreased in the acute phase. A future studies with a large sample size is needed for further understanding of the pathogenesis of VKH disease.

## References

[1] Moorthy RS, Inomata H, Rao NA (1995). Vogt-Koyanagi-Harada syndrome. Surv Ophthalmol.

[2] Bouchenaki N, Herbort CP (2001). The contribution of indocyanine green angiography to the appraisal and management of Vogt-Koyanagi-Harada disease. Ophthalmology.

[3] Lai T, Chan R, Chan C (2009). Effects of the duration of initial oral corticosteroid treatment on the recurrence of inflammation in Vogt-Koyanagi-Harada disease. Eye.

[4] Chee SP, Jap A, Cheung CM (2010). The prognostic value of angiography in Vogt-Koyanagi-Harada disease. Am J Ophthalmol.

[5] Hirahara S, Yasukawa T, Kominami A (2016). Densitometry of choroidal vessels in eyes with and without central serous chorioretinopathy by wide-field indocyanine green angiography. Am J Ophthalmol.

[6] Silva PS, Cavallerano JD, Haddad NM (2015). Peripheral lesions identified on ultrawide field imaging predict increased risk of diabetic retinopathy progression over 4 years. Ophthalmology.

[7] Domalpally A, Clemons TE, Danis RP, Writing Committee for the OPTOS Peripheral RetinA (OPERA) study (Ancillary Study of Age-Related Eye Disease Study 2) (2017). Peripheral Retinal Changes Associated with Age-Related Macular Degeneration in the Age-Related Eye Disease Study 2: Age-Related Eye Disease Study 2 Report Number 12 by the Age-Related Eye Disease Study 2 Optos Peripheral RetinA (OPERA) Study Research Group. Ophthalmology.

[8] Suetsugu T, Kato A, Yoshida M (2016). Evaluation of peripheral fundus autofluorescence in eyes with wet age-related macular degeneration. Clin Ophthalmol.

[9] Ogura S, Yasukawa T, Kato A (2014). Wide-field fundus autofluorescence imaging to evaluate retinal function in patients with retinitis pigmentosa. Am J Ophthalmol.

[10] Kawano Y, Tawara A, Nishioka Y (1996). Ultrasound biomicroscopic analysis of transient shallow anterior chamber in Vogt-Koyanagi-Harada syndrome. Am J Ophthalmol.

[11] Gohdo T, Tsukahara S (1996). Ultrasound biomicroscopy of shallow anterior chamber in Vogt-Koyanagi-Harada syndrome. Am J Ophthalmol.

[12] Kishi A, Nao-i N, Sawada A (1996). Ultrasound biomicroscopic findings of acute angle-closure glaucoma in Vogt-Koyanagi-Harada syndrome. Am J Ophthalmol.

[13] Kim AY, Rodger DC, Shahidzadeh A (2016). Quantifying retinal microvascular changes in uveitis using spectral-domain optical coherence tomography angiography. Am J Ophthalmol.

[14] Hirooka K, Saito W, Namba K (2015). Relationship between choroidal blood flow velocity and choroidal thickness during systemic corticosteroid therapy for Vogt-Koyanagi-Harada disease. Graefes Arch Clin Exp Ophthalmol.

